# Supplementary material for: Engineering and application of a biosensor with focused ligand specificity
Source: Nat Commun. 2020 Sep 25;11:4851. doi: 10.1038/s41467-020-18400-0 (PMC7519686; doi:10.1038/s41467-020-18400-0)
Supplement: Supplementary file 1 — Supplementary Information [file 41467_2020_18400_MOESM1_ESM.pdf]

Engineering and application of a biosensor with focused ligand specificity

Supplementary Information

Della Corte *et al.*

Supplementary Table 1 | Data collection and refinement statistics of LysG of *C. glutamicum*.

	LysG	LysG+Arg
**Data collection**		
Space group	P 41 21 2	P 41 21 2
Cell dimensions		
a, *b*, *c* (Å)	124.98 124.98 111.23	123.95 123.95 111.35
α, β, γ (°)	90 90 90	90 90 90
Resolution (Å)	49.94 - 2.52 (2.61 - 2.52)*	47 - 3.00 (3.11 - 3.00)*
Unique reflections	30335 (2992)	17846 (1752)
Multiplicity	9.6 (9.9)	7.2 (7.4)
Completeness (%)	99.92 (100.00)	99.65 (99.43)
Mean I/sigma(I)	25.02 (2.17)	15.77 (1.15)
Wilson B-factor	62.72	86.96
R-merge	0.06382 (1.206)	0.1299 (1.94)
R-meas	0.06746 (1.273)	0.1399 (2.084)
CC1/2	1 (0.742)	0.999 (0.428)
**Refinement**		
Resolution (Å)	49.94 - 2.52	47 - 3.00
No. reflections	30333 (2992)	17829 (1752)
R_{work} / R_{free}	0.1947 / 0.2274	0.2065 / 0.2508
No. atoms	4372	4383
Protein	4352	4371
Ligand/ion		12
Water	20	
B-factors	70.20	92.26
Protein	70.27	92.23
Ligand/ion		104.34
Water	53.97	
R.m.s. deviations		
Bond lengths (Å)	0.006	0.005
Bond angles (°)	0.82	0.86
PDB Accession Codes	6XTU	6XTV

*Values in parentheses are for highest-resolution shell.

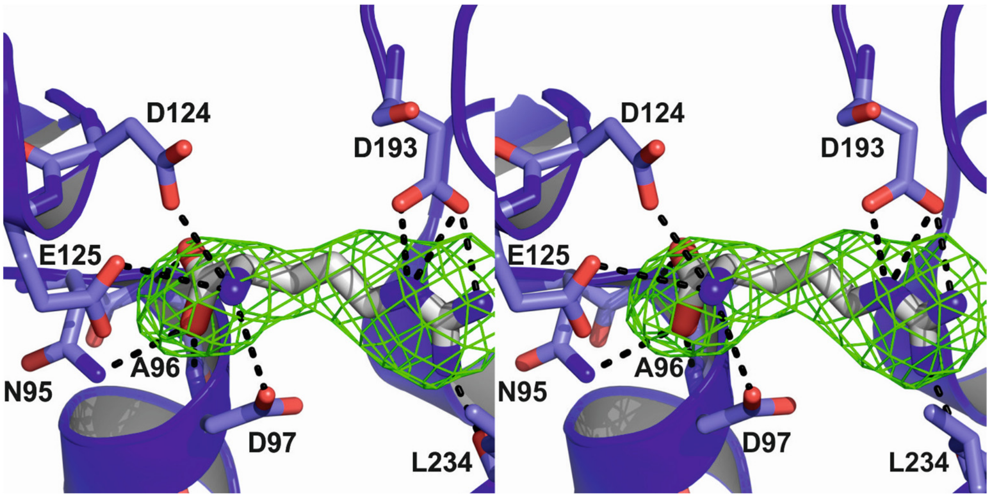

Supplementary Fig. 1 | Stereo view of the binding site of L-arginine in LysG. Shown is the binding site in the extended protomer B in the same orientation as in Fig1c in the main manuscript. Residues forming polar contacts closer than 3.6 Å are shown as sticks, with the contacts as broken lines. A simulated annealing omit FoFc map of L-arginine is shown as green mesh, contoured at 3σ.

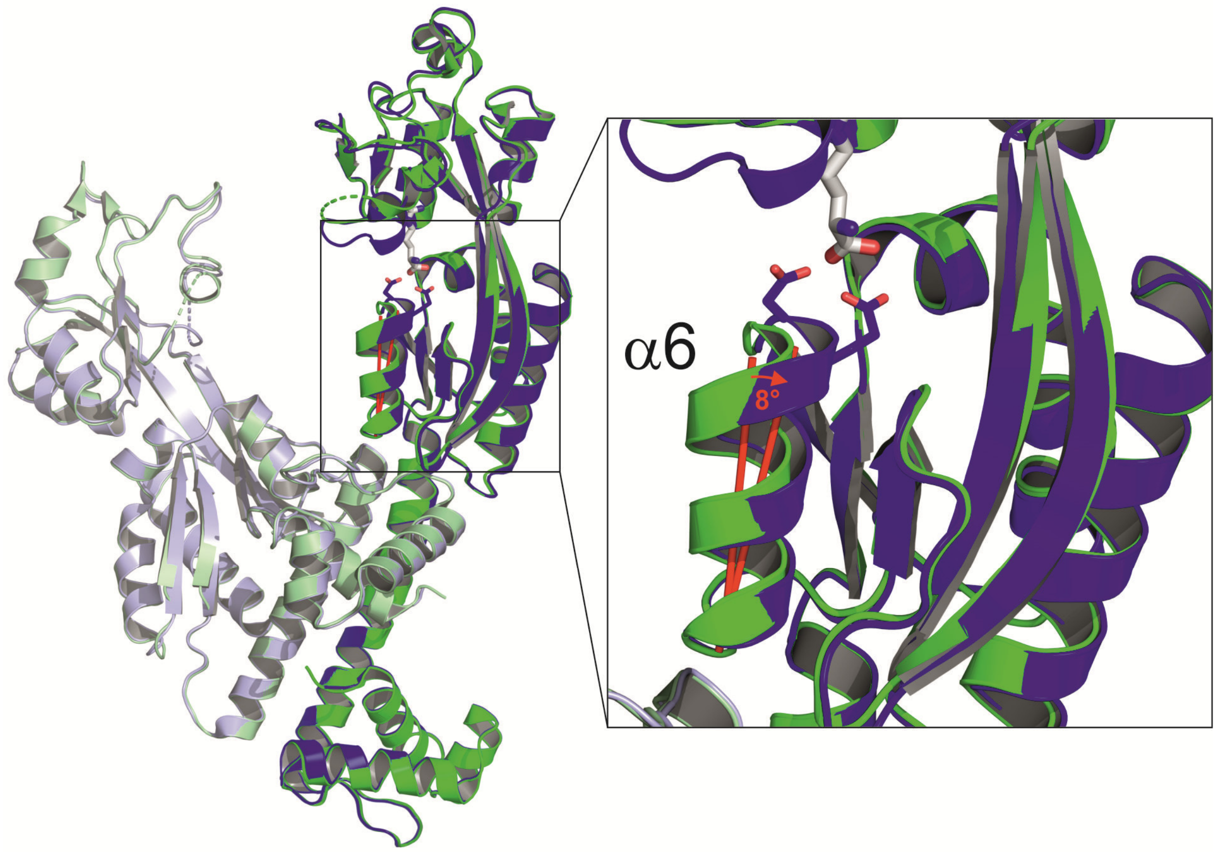

Supplementary Fig. 2 | Induced fit upon L-arginine binding to LysG. a, Superposition of the LysG dimer (green) and the LysG+Arg dimer (blue). The L-arginine free structure LysG is shown in green, the L-arginine bound structure LysG+Arg is shown in blue. Amino acid residues D124 and E125 in the LysG+Arg structure are pointing towards the ligand, thereby coordinating L-arginine. D124, E125 and L-arginine ligand (white) are shown as stick models. Oxygen atoms are shown in red, nitrogen atoms in blue. **b,** Zoom into the extended protomer's regulatory domain. The L-arginine-coordinating α-helix 6 (α6) of the extended protomer of LysG+Arg is tilted by 8° towards the inducer binding cavity. While in the crystal packing, no further domain movement is possible, in solution the interaction of α6 with the winged part of the winged helix-turn-helix (wHTH) motif of the compact protomer could lead to a modified DNA affinity. The protomers are shown in cartoon representation, the L-arginine molecule is shown as stick model and red lines illustrate the tilt of α6.

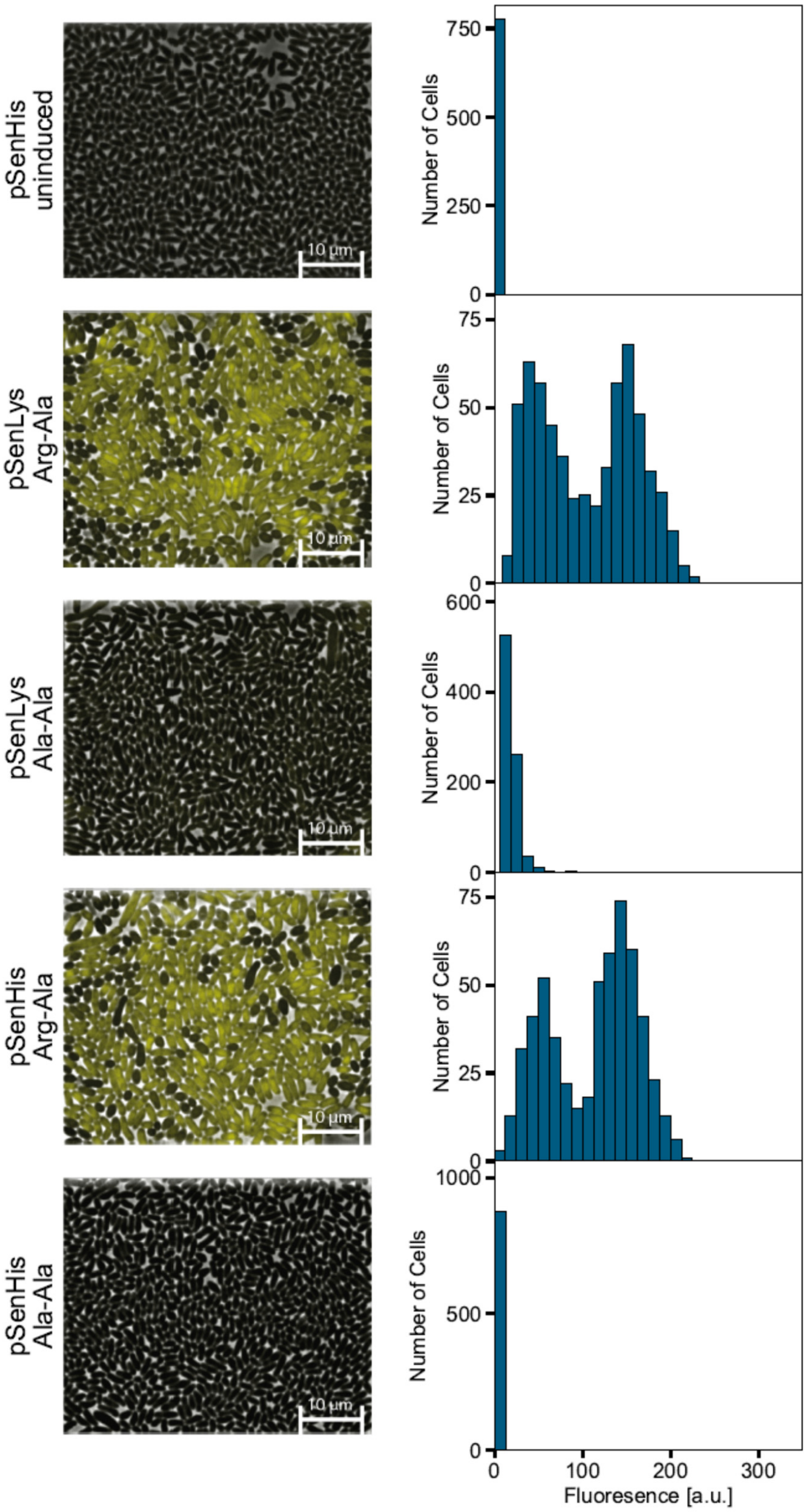

Supplementary Fig. 3 | Microscale cultivations of *C. glutamicum ΔlysEG* carrying pSenLys or pSenHis. Growth of corresponding cells in microfluidic chambers, on the left. Distribution of the fluorescence as measure of the biosensor response to the presence of L-Arg-L-Ala-dipeptides, L-Ala-L-Ala-dipeptides or no dipeptides at the single-cell level across microcolonies, on the right. Source data are provided as a Source Data file.

Supplementary Table 2 | Strains and plasmids used in this study.

Strain or plasmid	Relevant characteristics	Source,Reference
E. coli strains		
DH5α	F– Φ80*lacZ*ΔM15 Δ(*lacZYA-argF*)U169 *recA1 endA1 hsdR17* (rK–, mK+) *phoA supE44* λ– *thi-1 gyrA96 relA1*	Invitrogen (Karlsruhe, Germany)
BL21(DE3)	F^- *omp*T *hsd*SB (rB–, mB–) gal dcm rne131 (DE3)	Invitrogen (Karlsruhe, Germany)
C. glutamicum strains		
wild type	wild type strain, ATCC 13032, biotin-auxotroph	[1]
Δ*lysEG*	wild type strain with deletion of *lysE* (encoding the basic amino acid transporter LysE) and *lysG* (encoding the transcriptional regulator controlling *lysE* expression)	[2]
Plasmids		
pET28b(+)	*kan*^{R}; T7*lac* promoter; ColE1 replicon; *lacI;* 2x His_6-tag, T7-tag	Merck Millipore (Billerica, MA, USA)
pET28b(+)-*lysG*	pET28b(+) derivate for the heterologous expression of the *lysG* gene of *C. glutamicum* in *E. coli,* resulting in 20 additional N-terminal residues (His_6-Tag and Thrombin cleavage site).	this study
pET28b(+)-*lysG*-A219L	pET28b(+) derivate for the heterologous expression of the *lysG*-A219L variant in *E. coli*	this study
pEKEx3	*spec*^{r}; *E. coli - C. glutamicum* shuttle vector; *lacI*; P_{tac}; lacO1; pBL1 ori_{Cg}; pUC ori_{Ec}	[3]
pEKEx3-*lysG*	pEKEx3 derivate for expression of the *lysG* gene of *C. glutamicum*	this study
pSenLys	*kan*^{R}; *E. coli - C. glutamicum* shuttle vector; pHM1519 ori_{Cg}, pACYC177 ori_{Ec}. Biosensor plasmid for the detection of basic amino acids. Comprised of *lysG*, and its *lysE*-target promoter with a transcriptional fusion to *eyfp*	[4]
pSenHis	*kan*^{R}; *E. coli - C. glutamicum* shuttle vector; pHM1519 ori_{Cg}, pACYC177 ori_{Ec}. Biosensor plasmid for the detection of L-His and L-Arg. Comprised of the engineered *lysG*-A219L variant, and its *lysE*-target promoter with a transcriptional fusion to *eyfp*	this study

kan^r: kanamycin resistance, *spec*^{r}: spectinomycin resistance

Supplementary Table 3 | Oligonucleotides used in this study.

Oligonucleotide	Sequence (5´→ 3´)	Purpose
Fw-lysG-genome	gtgcaccatatgaaccccattcaactgga	Amplification of *lysG* from genomic DNA and subcloning into pET28b(+), *Nde*I-site
Rv-lysG-genome	ccgatgcttaagctaaaggccgcaatccctcgattg	Amplification of *lysG* from genomic DNA and subcloning into pET28b(+), *Eco*RI-site
Fw-pET28b(+)-seq	taatacgactcactataggg	pET28b(+) sequencing primer
Rv-pET28b(+)-seq	ctagttattgctcagcggt	pET28b(+) sequencing primer
Fw-LysG-N95X	cgttaaccatcgccatcNNSGCAGATtcgctatccac	saturation mutagenesis in *lysG* at position 95 (PCR in combination with Rv-LysG-R143X)
Rv-LysG-R143X	ccacgggattagcttcSNNggttaccgctcctaaaac	saturation mutagenesis in *lysG* at position 143 (PCR in combination with Fw-LysG-N95X)
Fw-LysG-D97X	catcgccatcAACGCANNStcgctatccacatggtttcc	saturation mutagenesis in *lysG* at position 97 (PCR in combination with Rv-LysG-H161X)
Rv-LysG-H161X	gttgcaatggccaaSNNgcgcatggttccaag	saturation mutagenesis in *lysG* at position 161 (PCR in combination with Fw-LysG-D97X)
Fw-LysG-D124X-E125X	cacgctgcgcttggaaNNSNNSgcgcacacattatccttg	saturation mutagenesis in *lysG* at positions 124 and 124 (PCR in combination with Rv-LysG-D124X-E125X)
Rv-LysG-D124X-E125X	caaggataatgtgtgcgcSNNSNNttccaagcgcagcgtg	saturation mutagenesis in *lysG* at positions 124 and 124 (PCR in combination with Fw-LysG-D124X-E125X)
Fw-LysG-F189X-D193X	gatgcccgtcttacgcNNSggtcccaaaNNSgtgcttcaagaccgtgacc	saturation mutagenesis in *lysG* at positions 189 and 193 (PCR in combination with Rv-LysG-F189X-D193X)
Rv-LysG-F189X-D193X	gtcacggtcttgaagcacSNNtttgggaccSNNgcgtaagacgggcatcg	saturation mutagenesis in *lysG* at positions 189 and 193 (PCR in combination with Fw-LysG-F189X-D193X)
Fw-LysG-G190X-P191X	gcccgtcttacgcttcNNSNNSaaagatgtgcttcaagaccgt	saturation mutagenesis in *lysG* at positions 190 and 191 (PCR in

		combination with Rv-LysG-G190X-P191X)
Rv-LysG-G190X-P191X	cggtcttgaagcacatctttSNNSNNgaagcgtaagacgggca	saturation mutagenesis in *lysG* at positions 190 and 191 (PCR in combination with Fw-LysG-G190X-P191X)
Fw-LysG-A219X-F222X	ccattgtcccgtcgNNSgaaggtNNSggtgaggcaattcgc	saturation mutagenesis in *lysG* at positions 219 and 222 (PCR in combination with Rv-LysG-A219X-F222X)
Rv-LysG-A219X-F222X	cgaattgcctcaccSNNaccttcSNNcgacgggacaatgga	saturation mutagenesis in *lysG* at positions 219 and 222 (PCR in combination with Fw-LysG-A219X-F222X)
Fw-lysG-seq	tgccggtggtgcagatgaactt	sequencing of *lysG* in SenLys/pSenHis
Rv-lysG-seq	gctccagatttatcagcaataaaccagccagccggaag	sequencing of *lysG* in SenLys/pSenHis
Fw-hisE-seq	cggtaaaaactctttcgaag	sequencing of genome-encoded *hisEG* genes
Rv-hisG-seq	tcctacgtacttgtaagagg	sequencing of genome-encoded *hisEG* genes
Fw-hisF-seq	tcgtttagtcctcatgtttg	sequencing of genome-encoded *hisFI* genes
Rv-hisI-seq	gatggttacccagttcatg	sequencing of genome-encoded *hisFI* genes
Fw-hisH-seq	ccacttgagagatgcttatc	sequencing of genome-encoded *hisHA* genes
Rv-hisA-seq	cttcggcatcatctacaac	sequencing of genome-encoded *hisHA* genes
Fw-hisD-seq	agtatctcaaaggtgaaagc	sequencing of genome-encoded *hisD* gene
Rv-hisD-seq	ttttcgttggtgttgagg	sequencing of genome-encoded *hisD* gene
Fw-hisC-seq	aattactttgagcgatttgc	sequencing of genome-encoded hisCB genes
Rv-hisB-seq	aaaccataaataaaccatcgg	sequencing of genome-encoded *hisCB* genes
Fw-hisN-seq	catcaaagtgaccgccggcg	sequencing of genome-encoded *hisN* gene

Rv-hisN-seq	gttgaggaaaattcgacca	sequencing of genome-encoded *hisN* gene

Capital letter indicate nucleobases, which are non-complementary to the template DNA. NNS-codons used for site-directed mutagenesis of selected codons in *lysG* encode for all 20 canonic amino acids and the TAG stop codon. N can be any of the four nucleobases, S represents G or C. All relevant recognition sites for endonucleases are underlined.

REFERENCES

1. Abe, S., Takayama, K. I. & Kinoshita, S. Taxonomical studies on glutamic acid-producing bacteria. *J. Gen. Appl. Microbiol.* **13**, 279–301 (1967).

2. Vrljić, M. *et al.* Expression control and specificity of the basic amino acid exporter LysE of Corynebacterium glutamicum. *Microbiology* **147**, 1765–1774 (2001).

3. Gande, R. *et al.* The two carboxylases of Corynebacterium glutamicum essential for fatty acid and mycolic acid synthesis. *J. Bacteriol.* **189**, 5257–64 (2007).

4. Binder, S. *et al.* A high-throughput approach to identify genomic variants of bacterial metabolite producers at the single-cell level. *Genome Biol.* **13**, R40 (2012).
