# Supplementary material for: Engineering and application of a biosensor with focused ligand specificity
Source: Nat Commun. 2020 Sep 25;11:4851. doi: 10.1038/s41467-020-18400-0 (PMC7519686; doi:10.1038/s41467-020-18400-0)
Supplement: Supplementary file 11 — Reporting Summary [file 41467_2020_18400_MOESM11_ESM.pdf]

nature research

Corresponding author(s): Jan Marienhagen

Last updated by author(s): Aug 11, 2020

Reporting Summary

Nature Research wishes to improve the reproducibility of the work that we publish. This form provides structure for consistency and transparency in reporting. For further information on Nature Research policies, see our Editorial Policies and the Editorial Policy Checklist.

Statistics

For all statistical analyses, confirm that the following items are present in the figure legend, table legend, main text, or Methods section.

n/a	Confirmed	
☒	☐	The exact sample size (*n*) for each experimental group/condition, given as a discrete number and unit of measurement
☐	☒	A statement on whether measurements were taken from distinct samples or whether the same sample was measured repeatedly
☒	☐	The statistical test(s) used AND whether they are one- or two-sided<br>*Only common tests should be described solely by name; describe more complex techniques in the Methods section.*
☒	☐	A description of all covariates tested
☒	☐	A description of any assumptions or corrections, such as tests of normality and adjustment for multiple comparisons
☐	☒	A full description of the statistical parameters including central tendency (e.g. means) or other basic estimates (e.g. regression coefficient) AND variation (e.g. standard deviation) or associated estimates of uncertainty (e.g. confidence intervals)
☒	☐	For null hypothesis testing, the test statistic (e.g. *F*, *t*, *r*) with confidence intervals, effect sizes, degrees of freedom and *P* value noted<br>*Give P values as exact values whenever suitable.*
☒	☐	For Bayesian analysis, information on the choice of priors and Markov chain Monte Carlo settings
☒	☐	For hierarchical and complex designs, identification of the appropriate level for tests and full reporting of outcomes
☒	☐	Estimates of effect sizes (e.g. Cohen's *d*, Pearson's *r*), indicating how they were calculated

Our web collection on statistics for biologists contains articles on many of the points above.

Software and code

Policy information about availability of computer code

Data collection	XDS and XSCALE (Ver.20141103) X-ray Detector Software - processing single-crystal monochromatic diffraction data recorded by the rotation method
Data analysis	Coot 0.8.9 (Crystallographic Object-Oriented Toolkit) - macromolecular model building of X-ray data, model completion and validation<br>Phenix 1.17. (Python-based Hierarchical ENvironment for Integrated Xtallography) software suite for the automated determination of molecular structures using X-ray crystallography data<br>SwissDock (no version data available) - web service to predict the molecular interactions that may occur between a target protein and a small molecule<br>FACSDiva 7.0.1 - used control the BD FACS device and to perform data analysis<br>FlowJo for Windows 10.4.2 - used to produce high-resolution graphics of FACS data<br>Prism 7.04 - used to produce high-resolution graphics of FACS data<br>NIS-Elements Microscope imaging software (Ver. 4.00) - analysis of cell areas and fluorescence values obtained from microfluidic time-lapse movies<br>MicroCal ITC analysis software (May 2015) - analysis of obtained Isothermal titration calorimetry-data<br>UCSF Chimera (1.13.1) - interactive visualization and analysis of molecular structures and related data<br>Dunbrack rotamer library - Conformational Analysis of Protein side chains, used in the context of MD modeling experiments<br>AutoDock (4.2.6) - suite of automated docking tools. It is designed to predict how small molecules, such as substrates or drug candidates, bind to a receptor of known 3D structure.

For manuscripts utilizing custom algorithms or software that are central to the research but not yet described in published literature, software must be made available to editors and reviewers. We strongly encourage code deposition in a community repository (e.g. GitHub). See the Nature Research guidelines for submitting code & software for further information.

April 2020

Data

Policy information about availability of data

All manuscripts must include a data availability statement. This statement should provide the following information, where applicable:

- Accession codes, unique identifiers, or web links for publicly available datasets
- A list of figures that have associated raw data
- A description of any restrictions on data availability

The authors declare that the data supporting the findings of this study are available within the paper and its supplementary information file. Macromolecular structural data were deposited to the Protein Data Bank (PDB) under accession codes 6XTU and 6XTV, respectively.

Field-specific reporting

Please select the one below that is the best fit for your research. If you are not sure, read the appropriate sections before making your selection.

☒ Life sciences ☐ Behavioural & social sciences ☐ Ecological, evolutionary & environmental sciences

For a reference copy of the document with all sections, see nature.com/documents/nr-reporting-summary-flat.pdf

Life sciences study design

All studies must disclose on these points even when the disclosure is negative.

Sample size	Generation of pairwise mutated LysG variants - Calculation of the individual library size was performed by plating 1/1000 of the respective mutagenesis batch on agar plates after transformation.<br>During microscale cultivations, the fluorescence of 750 cells was determined individually for each experiment
Data exclusions	No data was excluded
Replication	All cultivation experiments, ITC experiments and amino acid quantifications were performed in triplicates (stated in the text and in the materials and methods section)
Randomization	Randomization was not necessary - we knew the genetic constitution of all different biosensor variants isolated (Sanger sequencing). All subsequent characterizations were performed in triplicates
Blinding	After semi-rational mutagenesis we screened all clones - at this time-point we did not know the genetic constitution of the regulator gene in question.

Reporting for specific materials, systems and methods

We require information from authors about some types of materials, experimental systems and methods used in many studies. Here, indicate whether each material, system or method listed is relevant to your study. If you are not sure if a list item applies to your research, read the appropriate section before selecting a response.

Materials & experimental systems

n/a	Involved in the study	
☒	☐	Antibodies
☒	☐	Eukaryotic cell lines
☒	☐	Palaeontology and archaeology
☒	☐	Animals and other organisms
☒	☐	Human research participants
☒	☐	Clinical data
☒	☐	Dual use research of concern

Methods

n/a	Involved in the study	
☒	☐	ChIP-seq
☐	☒	Flow cytometry
☒	☐	MRI-based neuroimaging

Flow Cytometry

Plots

Confirm that:

☒ The axis labels state the marker and fluorochrome used (e.g. CD4-FITC).

☒ The axis scales are clearly visible. Include numbers along axes only for bottom left plot of group (a 'group' is an analysis of identical markers).

☒ All plots are contour plots with outliers or pseudocolor plots.

☒ A numerical value for number of cells or percentage (with statistics) is provided.

Methodology

Sample preparation	C. glutamicum pre-cultures were diluted to an optical density (OD600) of 0.5 in fresh CGXII medium containing 3 mM His-Ala. Equally treated precultures of C. glutamicum ΔlysEG carrying the biosensor plasmid pSenLys with the wild-type lysG gene, served as either positive control (+ 3 mM His-Ala) or negative control (no dipeptide supplementation) during all FACS-based screening and counterscreening experiments. For this purpose, all cultures were grown for 7 h at 30 °C and diluted to an OD600 < 0.1 in FACSFlow (BD) prior to fluorescence activated cell sorting. dipeptide.
Instrument	Single-cell autofluorescence analysis was performed using a FACSAria II (BD Biosciences, Franklin Lakes, NJ, USA) equipped with a 70 µm nozzle and run with a sheath pressure of 70 psi. A 488 nm blue solid laser was used for excitation. Forward-scatter characteristics (FSC) were recorded as small-angle scatter and side-scatter characteristics (SSC) were recorded as orthogonal scatter of the 488 nm laser. A 502 nm long-pass and 530/30 nm band-pass filter combination enabled EYFP fluorescence detection. Prior to data acquisition, debris and electronic noise were excluded from the analysis by electronic gating in the FSC-H against SSC-H plot.
Software	FACSDiva 7.0.1 - used control the BD FACS device and to perform data analysis
Cell population abundance	Using the fluorescence output of C. glutamicum ΔlysEG pSenLys induced with 3 mM His-Ala dipeptide (positive control), 200,000 cells characterized by similar or higher fluorescence were sorted into 5 mL reaction tubes (Eppendorf AG, Hamburg, Germany), prefilled with 3 mL fresh CGXII medium (positive sorting). After cultivation for two days at 30 °C, these cultures were used to inoculate fresh CGXII medium with 3 mM Lys-Ala dipeptide. Following a second cultivation for 7 h at 30 °C, cells were diluted in FACSFlow for single-cell autofluorescence measurements as outlined above. In contrast to the previous positive sorting, the fluorescence parameters of C. glutamicum ΔlysEG (pSenLys) grown in absence of dipeptide (negative control) was used to isolate cells without or with reduced fluorescence into fresh CGXII medium (negative sorting). The resulting cultures were subsequently subjected to an additional round of positive and negative sorting
Gating strategy	The six biosensor-libraries were combined (> 52,000 variants) and subjected to a FACS-based five-step screening/counter-screening strategy for identifying L-lysine-insensitive biosensor variants, still capable of eyfp-reporter gene expression upon recognition of L-histidine (see Fig. 2a in the main text! (not in the supplementary information). During this process, positive screening steps in the presence of 3 mM L-His-L-Ala-dipeptides for identifying L-histidine responsive biosensor variants (isolation of top 3% fluorescing cells) alternated with negative screening steps in the presence of 3 mM L-Lys-L-Ala dipeptides in which still-L-lysine-responsive biosensors were discarded (isolation of bottom 3% non-fluorescing cells). During this screening process, L-histidine responsive but L-lysine insensitive biosensor variants could be successfully enriched and 96 clones showing high fluorescence in the presence of L-His-L-Ala dipeptides were individually collected in a 96-well plate for subsequent analysis.

☒ Tick this box to confirm that a figure exemplifying the gating strategy is provided in the Supplementary Information.
